# Land Cover Change Intensifies Actual and Potential Radiative Forcing through CO_2_ in South and Southeast Asia from 1992 to 2015

**DOI:** 10.3390/ijerph16142460

**Published:** 2019-07-11

**Authors:** Yaoping Cui, Michael E Meadows, Nan Li, Yiming Fu, Guosong Zhao, Jinwei Dong

**Affiliations:** 1 Laboratory of Geospatial Technology for the Middle and Lower Yellow River Regions, Henan University, Kaifeng 475001, China; 2 Department of Environmental and Geographical Science, University of Cape Town, Rondebosch 7701, South Africa; 3 Institute of Geographic Sciences and Natural Resources Research, Chinese Academy of Sciences, Beijing 100101, China

**Keywords:** CO_2_ sequestration, warming, cooling, net ecosystem productivity, climate change

## Abstract

Land cover change (LCC) and its impact on CO_2_ sequestration and radiative forcing (RF) could dramatically affect climate change, but there has been little effort to address this issue in South and Southeast Asia over a long period of time using actual land cover information. In this study, annual land cover data from 1992 to 2015 were used to assess the CO_2_ flux and corresponding RF due to LCC in South and Southeast Asia. The results showed that 553.2 × 10^3^ km^2^ of the region experienced LCC during this period, mostly due to land reclamation, urban expansion, and deforestation. These LCC caused a marked net decrease in net ecosystem productivity (NEP) as a composite of the various land cover categories during the whole study period, especially since 2001. The CO_2_ sequestration was 2160 TgCO_2_ during the early 1990s however cumulative sequestration decreased by 414.95 TgCO_2_ by 2015. Correspondingly, the cooling effect of NEP, i.e. the total actual RF, was −0.366 W m^−2^ in South and Southeast Asia between 1992 and 2015. However, the potential RF of the cumulatively reduced NEP due to LCC relative to the 1990s resulted in a warming effect of 2.33 × 10^−3^ W m^−2^ in 2015. Our study provides an applicable framework to accurately assess the potential effect of large-scale LCC on climate.

## 1. Introduction

The impacts of land cover change (LCC) on CO_2_ must be accounted for when considering its mitigation effects in order to meet the Paris climate target [1]. LCC influences the amount of CO_2_ sequestered by terrestrial ecosystems through biogeochemical cycles and has been recognized as playing a significant role in global radiative forcing (RF) [2,3]. Numerous studies have demonstrated the profound effect of historical land cover on current and future climate [4,5]. Large-scale increase in global croplands (pastures) and a corresponding decrease in forest cover resulted in substantial CO_2_ emissions before 2000 and, indeed, CO_2_ emissions from LCC still account for approximately 10% of the total CO_2_ emissions in recent decades [6,7,8]. Importantly, changing land cover in a particular region can not only impact the climate over the world, but also impact the animal and plant habitats [9,10]. Moreover, climate change, especially temperature fluctuation, would increase the risks of public health.

CO_2_ is an important driver of climate change and will continue to be so in the future [11]. Extensive studies have been undertaken on changes in atmospheric CO_2_ concentration and their relationship to climate change across different timescales [12]. Nowadays, the effects of LCC on climate due to the change in CO_2_ has been analyzed in many studies [13,14,15]. However, although some of the above studies have taken into account the attenuation of CO_2_, the changing temporal patterns of total CO_2_ emissions have been neglected, as has the corresponding RF response affected by different integration years. In addition, most analyses have used simulated data in developing the scenarios with little research using actual land cover data in the corresponding period [8,16].

**Figure 1:**
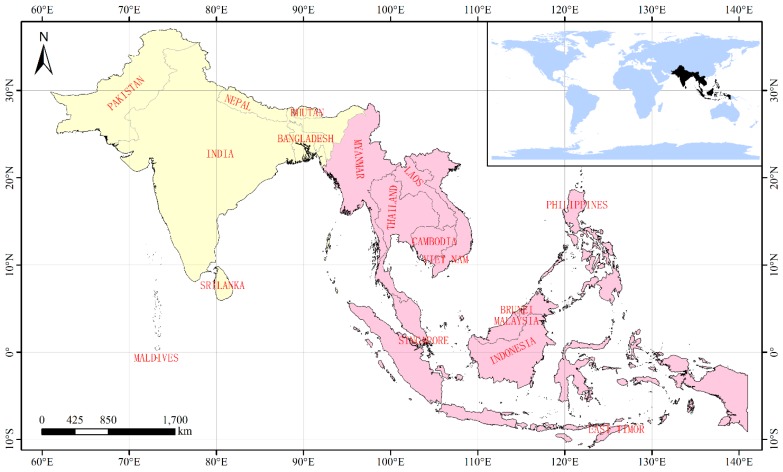
Area: South Asia (light yellow region) and Southeast Asia (light pink region).

South and Southeast Asia is comprised of the countries that lie between the tropics to the south of China (Figure 1), occupying a total area of 8.87 million km^2^. Since the 1990s, many countries such as India and Indonesia in South and Southeast Asia, have experienced a number of dramatic LCCs, including urban expansion, agricultural development, deforestation, and afforestation [17,18]. Previous studies have focused on the impact of LCC on the carbon cycle and evaluated its effects on carbon flux through various means, including ecosystem modelling, remote sensing, covariance observation, or using CO_2_ assimilation data [19,20]. However, such studies are usually limited to the analysis of one specific type of land cover conversion on the specific region [19,21]. Results thus far lack uniformity and there are discrepancies even when the eddy covariance type of measurement is used [20]. In addition, compared to the relatively complete CO_2_ flux network in China, North America, and Europe, there are only limited monitoring sites in South and Southeast Asia (AsiaFlux: http://asiaflux.net/?page_id=22) and these are too remote to analyze the CO_2_ flux of land cover categories in the region.

It is clear, therefore, that a detailed understanding of the influence of LCC on CO_2_ flux and RF in South and Southeast Asia is needed. The aim of this study is to develop a suitable framework to assess the RF response to the change of net ecosystem productivity (NEP) resulting from LCC between 1992 and 2015. Specifically, the objectives of this paper are as follows: (i) To obtain land cover categories and associated NEP values for each year between 1992 and 2015, (ii) to establish the benchmark NEP value, and (iii) to calculate the RF values of these years.

## 2. Materials and Methods

### 2.1. Study Area

The study area includes the two regions of South Asia and Southeast Asia (Figure 1). A total of 7 countries, Bangladesh, Bhutan, India, Maldives, Nepal, Pakistan, and Sri Lanka make up South Asia. The climate of these places varies from tropical monsoon in the south, to temperate in the north, a range which is influenced by altitude, proximity to the ocean, and the characteristics of the monsoon season. There are 11 countries in the region defined as Southeast Asia, namely, Brunei, Cambodia, East Timor, Indonesia, Laos, Malaysia, Myanmar, Philippines, Singapore, Thailand, and Vietnam. The climate is hot all year and rainfall is generally caused by the seasonal monsoon. Exceptions to this are found in Northern Vietnam and the Himalayan region, where higher elevations produce a cooler, subtropical climate (https://en.wikipedia.org/wiki/Main_Page).

### 2.2. Study Data

Data of 3 categories were collected for this study, specifically the annual land cover, terrestrial ecoregions, and NEP values for a suitable range of land cover categories. Annual land cover data at 300 m resolution for the period 1992 to 2015 are based on the Land Cover Map (v2.0.7; http://maps.elie.ucl.ac.be). The annual land cover data is also the only one that has been released to the public currently. The terrestrial ecoregions data were obtained from the map of terrestrial ecoregions of the world [22]. NEP data for each land cover categories were collated from current available literature.

**Figure 2:**
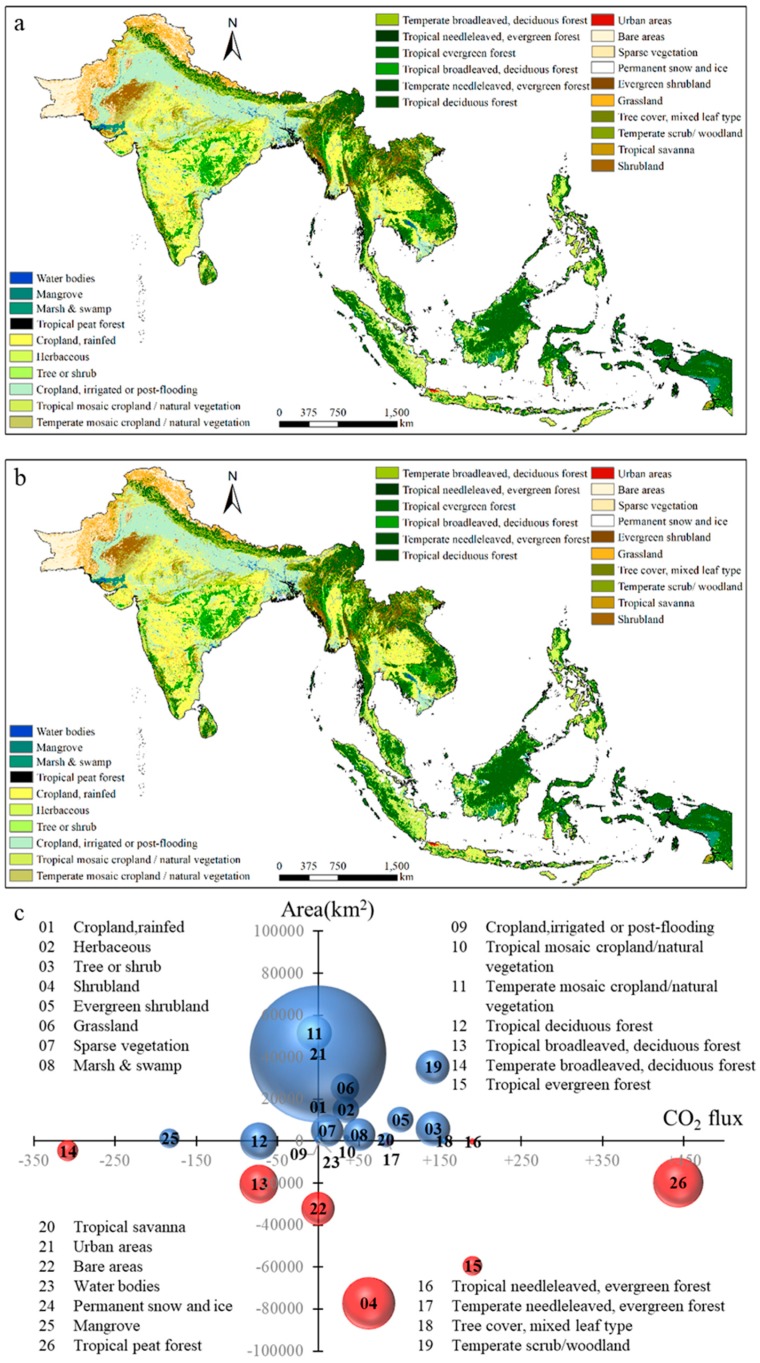
Land cover category change in study area. Spatial pattern of land cover categories in 1992 (**a**) and 2005 (**b**), and (**c**) temporal change of various land cover categories between 1992 to 2015. Bold and black numbers are the land cover categories; the size of circles represents magnitude of change, while their color indicates increase (blue) and decrease (red) of various categories.

In this study, with the annual land cover and terrestrial ecoregions data, the final land cover categories were defined based on plant functional categories (Figure 2) [23]. Based on previously published studies that considered the parameter of ecosystem-atmosphere exchange of CO_2_ in different terrestrial ecoregions, we determined NEP values for the various land cover categories and corresponding annual CO_2_ flux as shown in Table 1 (±NEP). All the NEP data were from public literatures.

### 2.3. Assessing the RF of Land Cover Category Change

In this analysis, we aimed to quantify the specific RF of LCC on an annual basis from 1992 to 2015. In order to assess the effects of terrestrial carbon uptake, firstly it was necessary to establish the regional benchmark quantity of CO_2_ uptake for the initial early 1990s conditions. In order to account for changes in net CO_2_ caused by land cover and to assess their effects on climate, an initial benchmark value is required against which the subsequent changes can be analyzed [33]. Here we used the mean NEP during 1992 to 1995 to represent the initial level of carbon sequestration (ICS) in the early 1990s. Thereafter, we compared the NEP of each of the subsequent years with the benchmark values. LCC is directly reflected in annual NEP values and the annual difference (AD amount) between NEP and ICS in each of the following years represents corresponding changes in the quantum of CO_2_ sequestration. Finally, we used a simplified climate response model to calculate the RF of NEP of terrestrial land cover from 1992 to 2015. There are many models that estimate the interactions between land cover, CO_2_, and RF [34]. However, considering the large uncertainties and discrepancies of their parameterizations and schemes, explicitly for illustrative purposes we adopted the widely-used empirical approach of Joos et al. (2013) to calculate RF [16,35].

(i) CO_2_ concentration. The changing NEP (*Ct* in tC) carried in various land cover categories or LCC can be converted to atmospheric CO_2_ concentration (*ACt* in ppm) by:

(1) $$AC_{t}=C_{t}/\left(2.213\times 10^{9}\right),$$

(ii) Actual RF (ARF). If the annual NEP > 0, then local land cover is acting as a carbon sink and therefore has a cooling effect, which can be expressed by the annual actual RF (ARF):

(2) $$ARF=-5.35\ln\left(1+abs(AC_{t}\right)/C_{0}),$$

where abs(f) is an absolute value function and *C_0_* is the background atmospheric CO_2_ concentration with a value of 354.39 in the early 1990s (www.esrl.noaa.gov/gmd/).

If the annual NEP < 0, then the annual warming effect of ARF can be calculated by:

(3) $$ARF=5.35\ln\left(1+abs(AC_{t}\right)/C_{0}),$$

(iii) Potential RF (PRF). Since natural ecosystems are in general carbon sinks and sequestrate CO_2_ from atmosphere, the concept of potential RF (PRF) is used here to express the potential climate effect of LCC.

If the annual difference between NEP and ICS is more than zero (AD > 0), local LCC results in carbon uptake and has a cooling effect. In this case, the exponential decay process of CO_2_ is ignored directly and only the first annual value of $AC_{t}$ is involved in the calculation of the same year. Or, if the AD < 0, there would be warming effect that compared with the ICS. Atmospheric CO_2_ at year t (t > 1991) can be calculated as:

(4) $$AC_{t}=\left[f_{0}+f_{1}\times exp\left(-\frac{t}{\tau_{1}}\right)+f_{2}\times exp\left(-\frac{t}{\tau_{2}}\right)+f_{3}\times exp\left(-\frac{t}{\tau_{3}}\right)\right]\Delta C$$

where *f_0_*, …, *f_3_* and *τ_1_*, …, *τ_3_* are the critical constants, usually used to define the global carbon cycle model [16,36]. Here we assign these parameter values based on the results of multi-mode simulations [16]. The changed *Ct* can be converted to added CO_2_ concentration ΔC (in ppm) by $\Delta C=\left(C_{t}-C_{0}\right)/\left(2.213\times 10^{9}\right)$. This formula accounts for the exponential decay process of CO_2_ [37].

Similar to the formula in Equation (3), the warming effect and the corresponding PRF due to LCC can be calculated by:

(5) $$PRF=5.35\ln\left(1+abs(AC_{t}\right)/C_{0})$$

## 3. Results

### 3.1. Land Cover and Land Cover Category Change

Currently, croplands and tropical evergreen forests are the two most abundant land cover categories in the region, each representing almost 20% of the study area (Figure 2). Croplands are most commonly found across India, Myanmar, Thailand, and Vietnam, while tropical evergreen forests are most prominently distributed in Malaysia, Indonesia, Brunei, Philippines, Cambodia, Vietnam, and other Southeast Asian countries. Land covered by cropland, either irrigated or naturally flooded, is distributed mainly in the northern part of India, accounting for 13% of the study area. The areas covered by mosaic cropland/natural vegetation in both tropical and temperate regions account for more than 5% of the total, with most of this land cover type existing in Southeast Asia.

Between 1992 and 2015, the land cover category changed across 553.2 × 10^3^ km^2^ of the region, and the most common conversions were urban expansion, land reclamation, and deforestation. The single largest proportion change in land cover was associated with urban expansion. The total amount of land classified as urban area in South and Southeast Asia increased by 41.5 × 10^3^ km^2^, representing a 1.72 fold increase from 1992 to 2015. Land cover categories, such as tropical mosaic cropland/natural vegetation, urban areas, and temperate scrub/woodland, expanded rapidly by increasing more than 25 × 10^3^ km^2^ for each type during the period in question. Tropical deciduous forest, tropical mosaic cropland/natural vegetation, and temperate scrub/woodland increased by more than 10%. Differently, shrubland, tropical peat forest, tropical evergreen forest, and tropical broadleaved, deciduous forest experienced the most significant decline, reducing in area by more than 18 × 10^3^ km^2^ for each type. Tropical peat forest and shrubland were both reduced by more than 20% over the time period.

### 3.2. Annual Variations in NEP

**Figure 3:**
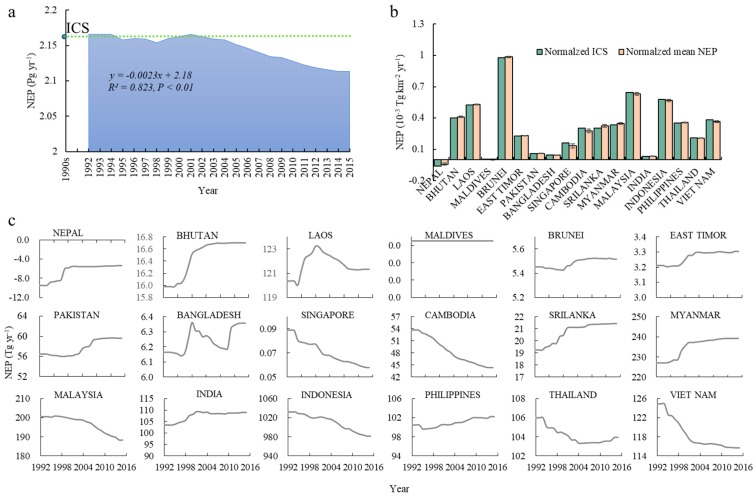
Values of NEP in the study area during 1992–2015. (**a**) Annual values of NEP in South and Southeast Asia; (**b**) annual values of NEP for all 18 countries; (**c**) initial level of carbon sequestration (ICS) and annual difference (AD) values of all 18 countries. The NEP of Maldives is zero due to its pocket land area.

For South and Southeast Asia as a whole, for the period 1992 to 2015, the values of ICS and mean annual NEP were 2.164 Pg and 2.146 Pg, respectively. The overall trend of NEP revealed a clear net decrease of 2.44 Tg yr^−1^ over the period (Figure 3a). In order to compare the ICS and NEP during 1992–2015 more effectively, we normalized the ICS and the multi-annual mean NEP for each country by converting the units to NEP per unit area. The normalized ICS and NEP for Brunei, Malaysia, Indonesia, and Laos all exceeded 0.5 × 10^−3^ Tg km^−2^ yr^−1^ (Figure 3b). The normalized multi-annual mean NEP values were greater than normalized ICS in 11 of the countries. In fact, the dynamics of the NEP also reflect this. With the exception of Nepal and Maldives, all 16 countries in South and Southeast Asia act as carbon sinks (Figure 3c). Most countries actually exhibited increased NEP relative to the benchmark between 1992–2015. However, because of substantial reductions in several of the larger countries, including Indonesia, Thailand, and Vietnam, the overall NEP values declined from 2.16 Pg in the early 1990s to 2.11 Pg in 2015 (Figure 3c). Indonesia had the largest NEP values, accounting for nearly half of the total region area as a whole and exhibited ICS and multi-annual averages of 1031.1 and 1010.1 Tg, respectively. Myanmar, Malaysia, Laos, India, Philippines, Thailand, and Vietnam all exceeded 100 Tg NEP annually. Among these, Myanmar, India, and Philippines all exhibited a trend of increase, while Cambodia, Malaysia, and Indonesia showed clear reductions.

**Figure 4:**
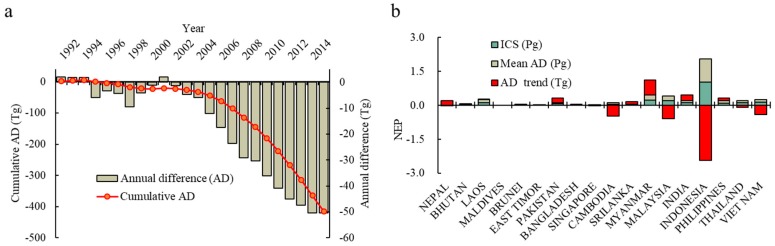
Dynamics of annual differences (AD) and cumulative AD over year (**a**); and the statistical information of AD and ICS in various countries (**b**).

In order to further evaluate the impact of LCC on NEP, we compared the AD of the annual recent NEP with the benchmark NEP values of the early 1990s (ICS). The results showed that the total AD of NEP was characterized by accelerated decreases (Figure 4a). When annual NEP is compared with ICS, AD during 1992–2002 remained around −2.0 Tg, with a range of −9.54 to 2.02 Tg. Thereafter, the AD increased and, by 2015, reached 50.41 Tg, indicating that the overall ability of the land cover of the region to sequester CO_2_ was declining year by year.

Over the study period, the quantum of cumulative carbon sequestration decreased by 414.95 Tg compared with the early 1990s. Among the 18 countries, the largest contributors to this change were Indonesia (503.5 Tg), Vietnam (139.3 Tg), Cambodia (118.8 Tg), and Malaysia (94.2 Tg). In fact, the amount of NEP in some countries, such as Myanmar, India, Nepal, and Laos increased over a year (Figure 4b). However, the total increased amount of NEP was too far to offset the substantial decreasing amount of NEP in several major countries. The result was also mutually confirmed with the point we obtained above.

### 3.3. RF of Land Cover and Land Cover Category Change

**Figure 5:**
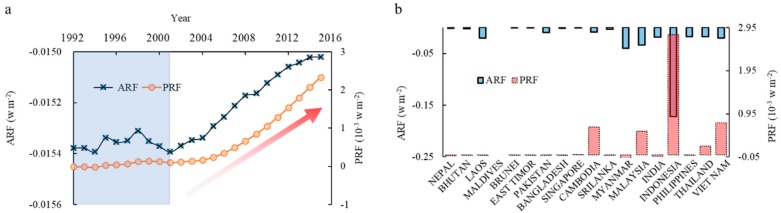
Actual radiative forcing (ARF) and potential radiative forcing (PRF) in South and Southeast Asia (**a**) and 18 countries (**b**).

Land cover change intensifies radiative forcing. From the perspective of global temperature change, ±RF represents the warming or cooling effect due to the annual change of NEP or AD. The results show that, while NEP remained positive in the region, the AD was negative in almost all 18 countries. This indicates that the climate effect of terrestrial ecosystems of South and Southeast Asian countries from 1992 to 2015 was cooling overall however the magnitude of this effect is declining with time due to LCC. Analysis of the temporal and spatial dynamics of annual actual radiative forcing (ARF) during 1992 to 2015 and potential radiative forcing (PRF) in 2015 is shown in Figure 5. Annual ARF changes are similar to trends in NEP in that, during 1992 to 2015, values declined by a total of −0.366 W m^−2^. However, the annual negative ARF had a shapely increase trend, especially since 2002, meaning that the capacity of carbon sequestration by terrestrial land cover categories in the study area decreased over time (Figure 5a). Unlike ARF, PRF continued to rise because it considers the cumulative RF across the whole study period (Figure 5a). Compared to the ICS, the PRF in 2015 reached 2.331 × 10^−3^ W m^−2^. In essence, this means that the NEP reduction caused by LCC during 1992 to 2015 yielded a warming effect of 2.331 × 10^−3^ W m^−2^ to 2015. In particular, Figure 5a clearly shows that both ARF and PRF exhibited a very significant linear increase since 2001.

In general, less negative values of ARF induce pronounced cooling effects, correspondingly, larger positive PRF values induce stronger warming effects due to LCC, which may be intensified or attenuated by other anthropogenic and/or natural factors. ARF values for all countries in the region were negative (Figure 5b), indicating that all the countries made a general contribution to the cooling effect, especially Indonesia, Myanmar, and Malaysia, which have mean ARF values below −0.3 W m^−2^, such that these countries play a role in global temperature attenuation. In terms of PRF, six countries exhibited positive values, namely Indonesia, Vietnam, Cambodia, Malaysia, Thailand, and Singapore, showing that LCC in these countries markedly impacted climate through an accentuated warming effect. In addition, in terms of the climate effect of various land cover types, urban area expansion and deforestation in particular, the area shrinking of tropical peat forest and tropical needle leaved evergreen forest contributed the most in the whole study area with 90.26% shares on the total RF increment during the study period.

## 4. Discussion

### 4.1. Uncertainty Analysis

There are inherent problems in assessing the accuracy of estimating terrestrial productivity [38,39]. While accurate assessment of the net CO_2_ emissions arising from LCC is a fundamental research goal, unfortunately there remains considerable uncertainty in such estimates. A meta-analysis of 250 studies of carbon responses to land cover change revealed conflicting results for several land cover transitions, with some resulting in net carbon uptake (for example agriculture to forest) while others involved carbon release (mature forest to agriculture) [40]. In the present study, because the land cover classification used is based mainly on the functional characteristics of terrestrial vegetation, the land cover categories identified may not necessarily correspond to each other in regard to CO_2_ flux. Additionally, the situation of widely distributed cropland and forest ecosystem is complex. Photosynthesis and subsequent harvesting, biomass burning, deforestation, degradation, and other greenhouse gases like N_2_O have not been considered [41]. This means that the study does not account for the effects of all greenhouse gases from these land cover categories and simplifies the NEP changes within the same land cover category. This also implies that CO_2_ emissions due to deforestation and CO_2_ uptake due to regrowth may be underestimated in this study. However, our results showed that the NEP during the 1990s was 589.1 TgC yr^−1^, which is similar to the result (546.6 TgC yr^−1^) obtained by Cervarich et al. (2016). As for the flux, results differ widely between studies [38,42]. Other studies reveal NEP values that are much higher than our results, implying that considering only the land cover category change may underestimate the climate regulation effect of LCC to some extent. To further explore the specific results of this study, we can compare several basic ratios with global results for reference. The study area accounts for 8.87/149 = 5.95% of the global continental land mass. For the NEP and RF, for reference, the annual values of NEP during 2006–2015 and RF during 2003–2012 were calculated to compare the global results with Global Carbon Project and other studies. The ratios of NEP and RF reached 9.28 (103/1110) and 6.98%, respectively [43,44]. The fact that these ratios are greater than their respective areas indicates the importance of regional factors in regulating climate change.

The CO_2_ flux between vegetation and soil is not synchronized. For example, the annual carbon release from soil varies until the soil is “set” to another type of land cover completely. For this issue, some studies introduced a bookkeeping approach to address which calculations were based on stocks of carbon in vegetation and soils but not the CO_2_ fluxes for ecosystems [13,24,45]. Although carbon stocks are relatively fixed and using CO_2_ flux during 1992–2015 can sensitively capture the resultant RF, carbon stocks can be used to calculate a long-term result, which can completely consider the full decomposition of various biomasses or ecosystems. Overall, although the accuracy of the study data and methods themselves may be constrained, the calculated result and framework are suitable for assessing the relative changes in the nature and distribution of changes in RF.

### 4.2. Problem and Prospects

Key results obtained in this study are noteworthy. This study was undertaken on the basis of a carbon balance, which considered the initial condition (benchmark) of the early 1990s to be NEP = 0, enabling subsequent changes in the NEP and the corresponding RF variations to be effectively evaluated. The results reveal that, although total NEP in South and Southeast Asia remained positive during 1992–2015, CO_2_ sequestration by various land cover categories declined substantially, and the cooling effect of NEP in the region declined over the study period. This situation emerged directly from the corresponding LCCs. The Southeast Asian tropical rainforest is the second largest tropical rainforest on earth, with data revealing that the area under this land cover category has continued to decline during the period in question [39]. Coupled with urban expansion and increased farmland conversion, NEP has been reduced with concomitant climate implications, as indicated.

In contrast to previous studies that used simulated data and numerical models, our analysis of the effect of NEP on RF employed actual annual land cover data during 1992–2015. The simplified parameterization scheme used in this study not only facilitated the use of actual land cover data, but also avoided the constraints of numerical models based on the explicit correspondence between land cover and RF in the aspect of biogeochemistry. It was confirmed that the results of the single general circulation model (GCM) often have very large discrepancies and the Coupled Model Intercomparison Project (CMIP) are thus used by IPCC to analyze the RF and climate effects of greenhouse gases [16,34]. In fact, based on the multi-mode simulations, simplified climate response models are generally parameterized to reflect the main characteristics of GCMs and these methods have been used extensively by IPCC. The analysis here enables the direct interpretation of RF changes corresponding to CO_2_. In addition, many studies commonly assess the climate effects of CO_2_ on the basis of a comparison with a pre-industrial revolution atmosphere and, in the process, integrate their analyses over a period of 100 years or more [36,46]. The global atmospheric CO_2_ concentration was 278.00 ppm in 1750 compared to more than 350 ppm in the 1990s. The assessment results based on 1990s actually indicated a contribution since 1990 for future temperature. Furthermore, in the process of data analysis, annual CO_2_ emissions are important for calculating the RF since it can consider the real-time situation. However under the condition whereby the total amount of emitted CO_2_ is fixed, a different emission time or emission scenario will also affect its CO_2_ concentration at a certain year, and then affect the subsequent RF [16,37].

However, given that the simplified carbon-climate parameterization scheme cannot consider biomass burning, black carbon, and other factors associated with LCC, a full evaluation of the radiative forcing in South Asia/Southeast Asia remains elusive, although the climate effect of CO_2_ can be analyzed. In this study, NEP, the actual, and potential RF (ARF and PRF) were used to construct a bookkeeping framework for a preliminary assessment of the climate effect of LCC. In order to improve the assessment approach, two aspects should at least be considered in future to more finely resolve the climate effects of LCC. One issue is the temporal scale. Limited by the formula and analysis period, the later in the sequence that the LCC occurs, the lower its effect because the remaining amount of CO_2_ decay is not computed after 2015. This study was strictly stuck in the study period. A second issue concerns the framework itself. Although CO_2_ is the main greenhouse gas and the focus of this study, other greenhouse gases, including CH_4_ and N_2_O, are affected by LCC [24,36]. The climate effect of LCC also involves biogeophysical processes. Changes emanating from variations in land cover are well correlated with spatial patterns in surface biogeophysical parameters, such as albedo, evapotranspiration, surface roughness, etc. [13,47,48]. LCC represent an even stronger driver of climate change when taking into account biogeophysical mechanisms and biogeochemistry aspects at the local or regional scale [8]. Therefore, considering the basic assessment framework utilized in this study, other related work on the climate effect of LCC should be conducted in the future.

## 5. Conclusions

This study used actual land cover data in South and Southeast Asia from 1992 to 2015 to assess actual and potential radiative forcing of CO_2_ due to land cover category change in South and Southeast Asia from 1992 to 2015. Although the bookkeeping approach adopted does not account for other complex biogeochemical and biogeophysical effects, our framework enables a rapid assessment on the climate effects of LCC. Results of this preliminary assessment indicate that the ability to sequestrate CO_2_ was weakened due to LCC during 1992 to 2015, resulting in a net warming effect of 2.33 × 10^−3^ W m^−2^ to 2015. In particular, both ARF and PRF exhibited a very significant linear increase since 2001. Overall, the presented results could contribute to other sciences in the broader perspectives, especially climate change and its effects on the living world.

## Figures and Tables

**Table 1:** Atmosphere exchange of CO_2_ of each undisturbed land cover type.

Land Cover Type	**CO_2_** Flux ^a^	References ^b^
Cropland, rainfed	0	[13,24]
Herbaceous	+34	[13,24]
Tree or shrub	+141	[13,24]
Cropland, irrigated or post-flooding	0	-
Tropical mosaic cropland/natural vegetation	+35	[13,24]
Temperate mosaic cropland/natural vegetation	−5	[13,24]
Tropical evergreen forest	+190	[13]
Tropical broadleaved, deciduous forest	−73	[13]
Temperate broadleaved, deciduous forest	−308	[13,25,26]
Tropical needle leaved evergreen forest	+190	[13,26]
Temperate needle leaved, evergreen forest	+86	[13,26]
Tropical deciduous forest	−73	[13,26]
Tree cover, mixed leaf type	+155	[13,24,26]
Temperate scrub/woodland	+141	[24]
Tropical savanna	+83	[13,24]
Shrubland	+62	[27]
Evergreen shrubland	+101	[28]
Grassland	+33	[13,24,26]
Sparse vegetation	+11	[13,24]
Marsh and swamp	+50	[29]
Urban areas	0	-
Bare areas	0	-
Water bodies	0	-
Permanent snow and ice	0	-
Mangrove	−183	[30]
Tropical peat forest	+443	[31,32]

Note: ^a^ positive (+) and negative (−) values represent the CO_2_ uptake and release of various undisturbed land cover types, respectively. Units: kmol ha^−1^ yr^−1^. ^b^ “-” means that there is no available references for the net ecosystem productivity (NEP) value.
